# A Rubber-Tapping Robot Forest Navigation and Information Collection System Based on 2D LiDAR and a Gyroscope

**DOI:** 10.3390/s19092136

**Published:** 2019-05-08

**Authors:** Chunlong Zhang, Liyun Yong, Ying Chen, Shunlu Zhang, Luzhen Ge, Song Wang, Wei Li

**Affiliations:** College of Engineering, China Agricultural University, Qinghua Rd.(E) No.17, Haidian District, Beijing100083, China; zcl1515@cau.edu.cn (C.Z.); yly562468@163.com (L.Y.); zhangshunlu@cau.edu.cn (S.Z.); luzhenge@cau.edu.cn (L.G.); 18811575767@163.com (S.W.); liww@cau.edu.cn (W.L.)

**Keywords:** 2D LiDAR, gyroscope, navigation, information collection, map construction

## Abstract

Natural rubber is widely used in human life because of its excellent quality. At present, manual tapping is still the main way to obtain natural rubber. There is a sore need for intelligent tapping devices in the tapping industry, and the autonomous navigation technique is of great importance to make rubber-tapping devices intelligent. To realize the autonomous navigation of the intelligent rubber-tapping platform and to collect information on a rubber forest, the sparse point cloud data of tree trunks are extracted by the low-cost LiDAR and a gyroscope through the clustering method. The point cloud is fitted into circles by the Gauss–Newton method to obtain the center point of each tree. Then, these center points are threaded through the Least Squares method to obtain the straight line, which is regarded as the navigation path of the robot in this forest. Moreover, the Extended Kalman Filter (EKF) algorithm is adopted to obtain the robot’s position. In a forest with different row spacings and plant spacings, the heading error and lateral error of this robot are analyzed and a Fuzzy Controller is applied for the following activities: walking along one row with a fixed lateral distance, stopping at fixed points, turning from one row into another, and collecting information on plant spacing, row spacing, and trees’ diameters. Then, according to the collected information, each tree’s position is calculated, and the geometric feature map is constructed. In a forest with different row spacings and plant spacings, three repeated tests have been carried out at an initial speed of 0.3 m/s. The results show that the Root Mean Square (RMS) lateral errors are less than 10.32 cm, which shows that the proposed navigation method provides great path tracking. The fixed-point stopping range of the robot can meet the requirements for automatic rubber tapping of the mechanical arm, and the average stopping error is 12.08 cm. In the geometric feature map constructed by collecting information, the RMS radius errors are less than 0.66 cm, and the RMS plant spacing errors are less than 11.31 cm. These results show that the method for collecting information and constructing a map recursively in the process of navigation proposed in the paper provides a solution for forest information collection. The method provides a low-cost, real-time, and stable solution for forest navigation of automatic rubber tapping equipment, and the collected information not only assists the automatic tapping equipment to plan the tapping path, but also provides a basis for the informationization and precise management of a rubber plantation.

## 1. Introduction

As an essential strategic resource, natural rubber and its products are universally applied in industry, transportation, national defense, and medical treatment. At present, the excellent properties of natural rubber cannot be exceeded by synthetic materials [1,2,3,4]. The demand for natural rubber products keeps increasing with the development of economies and industries [5]. Currently, the rubber-tapping activity is still dominated by manual work both at home and abroad [6]. Due to the labor’s intensity and technical difficulty, the poor working environment, the shortage of workers, and aging in the working population, there is a sore need for intelligent rubber-tapping devices [7,8]. The autonomous navigation technique is of great importance to make rubber-tapping devices intelligent, especially in night-time operation.

In addition, the diameter, plant spacing, and row spacing of trees are important parameters for evaluating orchards or forests. The same is true of rubber plantations. These parameters reflect the growth status, water consumption, and biomass of rubber trees. Accurate and up-to-date mapping and monitoring of a rubber plantation are challenging [9,10].

To date, many technologies, such as sensors, RTK-GPS (RTK: Real-time kinematic; GPS: Global Positioning System), machine vision, LiDAR, and ultrasonic geomagnetic position, have been developed to study the autonomous navigation of robots [11,12]. However, machine vision is affected by the working environment and lighting conditions to a large extent, and technologies involved in it, such as image processing, image analysis, camera calibration, and the extraction of navigation parameters, make it rather difficult to apply in agriculture [13,14,15,16]. The application of GPS is affected by interruptions in the satellite signal [11,17]. For example, when a vehicle travels through a forest with too many trees, the crown often blocks the signals sent by satellites to GPS receivers, while RTK-GPS, with higher precision, is too expensive to be widely used [9,18]. Rubber-tapping techniques require this activity to be carried out at night. LiDAR can not only provide a large amount of accurate information with high frequency, but also can meet the accuracy and speed requirements. Moreover, with great performance compared to cost, it provides all-weather services regardless of lighting conditions [19]. Therefore, it has been widely applied to robot navigation and positioning as well as map construction in non-agricultural fields [20,21,22,23], and is becoming increasingly popular in the navigation of agricultural vehicles [24,25,26,27].

Benet [18] and his colleagues employed LiDAR, an Inertial Measurement Unit (IMU), and a camera sensor in their study. The environmental–geometric data collected by the IMU are obtained with the color camera’s data in order to improve the efficiency of object recognition. Then, by sorting the colors, objects in front of the vehicle, such as crops, grass, leaves, and soil, are inspected and recognized. Thus, agricultural vehicles can detect obstacles and navigate along crop rows autonomously. Similarly, in the paper of [24,25], by fusing data from LiDAR, a camera, and an IMU, the trunks of trees were detected and a partial map of an orchard was thus constructed. The Kalman Filter algorithm was also used to estimate the robot’s position in this orchard, so as to make it navigate autonomously among tree rows. Shalal [26] et al. proposed a new method for local-scale orchard mapping based on tree trunk detection and utilizing modest-cost color vision and laser-scanning technologies. The image segmentation and data fusion methods were used for feature extraction, tree detection, and orchard map construction. The map is composed of the coordinates of the individual trees in each row as well as the coordinates of other non-tree objects, and it can be used as an a priori map to realize robot positioning and path planning and navigation in the orchard. However, the application of the camera increases the complexity and the amount of calculation of the system. In the papers of [27,28], taking LiDAR as the navigation sensor, Barawid et al. have made it possible for the robot to navigate autonomously along a particular tree row of the orchard. Due to the lack of attitude information, the number of navigation errors will increase if the robot walks on uneven ground. Additionally, the turning performance of the robot has not been studied. Therefore, the key to increasing the precision and robustness of a navigation system lies in the combination of LiDAR and Inertial Measurement Units (IMUs) [19,29]. Bayar [30] et al. have made full use of the data from LiDAR, wheels, and the steering encoder. A motion model, including wheel side slip, was constructed to calculate the vehicle’s speed and its steering instructions. It can not only autonomously navigate among rows in the orchard, but also can generate the turning path and turn at the end of the row. Compared with the expensive RTK-GPS, this low-cost sensor suite is of much more commercial value. However, turning at the end of the rows is probabilistic, so the success of each turn cannot be guaranteed. Moreover, the automatic rubber-tapping operation requires the robot navigation platform to safely walk along one row in a rubber forest, to turn from one row into another, and to stop within a fixed range for tapping. Freitas [31] et al. have applied LiDAR, an IMU, a steering wheel, and wheel encoders to assess the position of offline obstacles based on the classification and clustering of three-dimensional (3D) point obstacles. Finally, obstacles, such as pedestrians and boxes, on the route of the vehicle have been detected successfully. To predict the biomass, growth, yield, water consumption, and health conditions of trees so as to provide bases for tree management, Lee [32] et al. have successfully adopted LiDAR to measure the geometric characteristics of tree crowns. The results show that LiDAR can better measure the geometric characteristics of the tree.

In summary, due to the excellent comprehensive performance of LiDAR, a method of forest navigation and information collection based on low-cost LiDAR and a gyroscope, which does not rely on GPS, is proposed in this paper. The sparse point cloud data of tree trunks are extracted by LiDAR and the gyroscope through the clustering method. The point cloud is fitted into circles by the Gauss–Newton method to obtain the center point of each tree. These center points are threaded through the Least Squares method to obtain the straight line, which is regarded as the navigation path of the robot in this forest. The Extended Kalman Filter (EKF) algorithm is adopted to obtain the robot’s position. The Fuzzy Controller is applied for the following activities: walking along one row with a fixed lateral distance, stopping at fixed points, turning from one row into another, and collecting the information of plant spacing, row spacing, and trees’ diameters. Then, according to the collected information, each tree’s position is calculated, and the geometric feature map is constructed. The aim of the method proposed in this paper is to provide autonomous navigation for intelligent tapping devices and collect information on a rubber plantation to benefit the informatization and precision management of the rubber plantation.

## 2. Materials and Methods

### 2.1. System Composition

**Figure 1:**
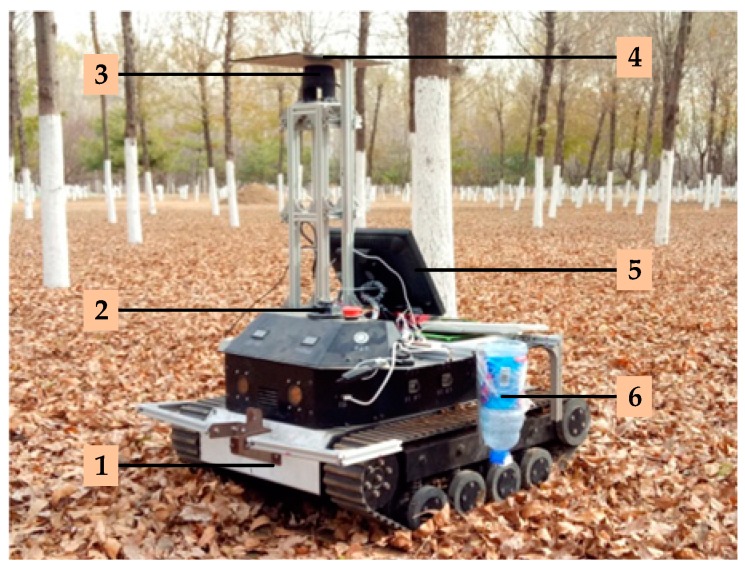
The Robot System. 1. Car body; 2. Gyroscope; 3. LiDAR; 4. Shading board; 5. Display screen; 6. Tracing device.

**Figure 2:**
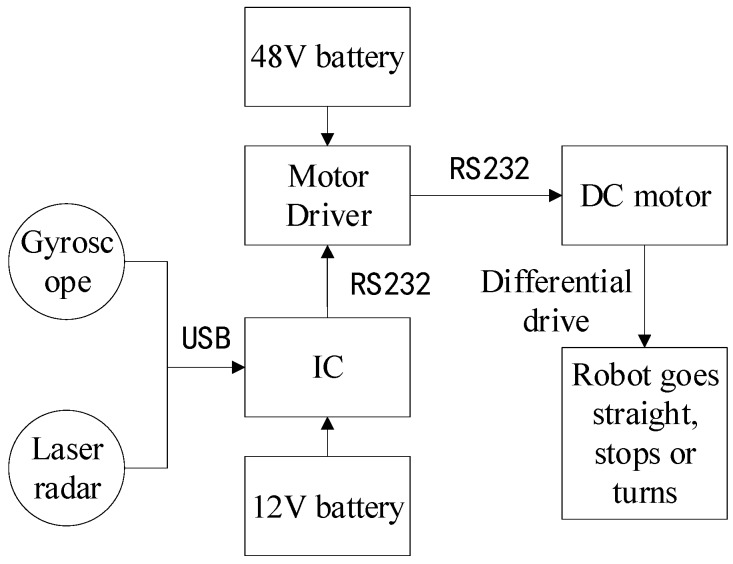
The principle of the system.

This study takes a tracked robot as the basic platform, the system of which is shown in Figure 1. The program runs on Windows 7 Visual Studio 2013. The length of the robot platform is 76 cm and the width is 62 cm. The distance between the LiDAR’s installation site on the robot platform and the ground is 95 cm. The type of this LiDAR is A2M6; it is made by SLAMTEC Company, and has a scanning frequency of 10 Hz, a scanning angle of 360 degrees, an angle resolution that is adjustable from 0.45 to 1.35 degrees, a maximum scanning distance of 18 m, and a relative scanning accuracy of 1%. The gyroscope used in this study is of type JY901 and produced by Shenzhen Witt Intelligent Technology Co., Ltd. Its frequency is 10 Hz, and its angular resolution is 0.1 degrees. Two caterpillar driving wheels are driven by two HLM480E36LN brushless direct current (DC) motors made by MOTEC Company. The role of the trace device: white wheat flour is placed in the tracing device, so when the robot walks, the white line left on the ground should be the actual walking path of the robot. As shown in Figure 2, the motor is directly controlled by the industrial computer (IC) through its driver. The Industrial Computer is the control core of the robot. 

### 2.2. Navigation Strategy

For targeting under specific conditions of rubber-tapping, mobile robots are required to be able to stop in front of trees and work. So, the robot platform set should achieve the following goals in the forest:

- Autonomously navigate along one row with a fixed lateral distance;
- Stop at the designed spot in front of trees;
- Turn from one row into another;
- Information collection.

**Figure 3:**
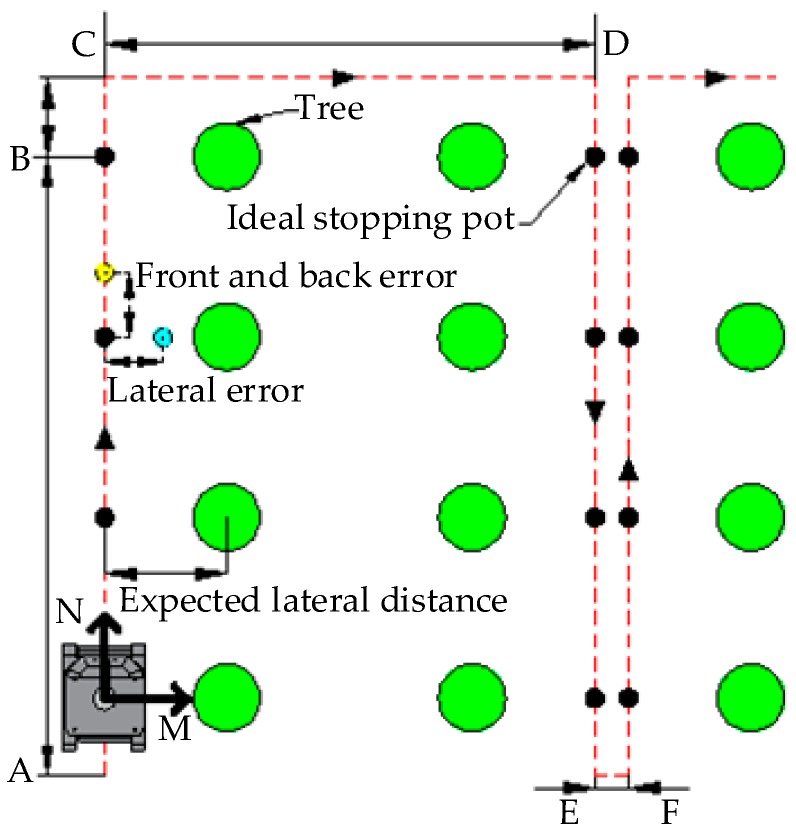
The navigation path.

According to the trend of trees, the spot that crosses the tree’s center and is perpendicular to the tree row is regarded as dead ahead of a tree. As required by the rubber-tapping operation of the mechanical arm, the robot’s ideal stopping spot should be 125 cm right ahead the rubber tree. Therefore, the ideal navigation path for the robot in a rubber forest should be the line chart that connects the dead ahead spot of each tree. In order to fit tree rows better, the segmentation fitting method is thus proposed. At the same time, the LiDAR scanning distance is set to be twice the maximum plant space to make full use of the tree data closest to the robot. If there are no data scanned within 0~90° of the LiDAR, it is supposed that the robot has reached the boundary of this rubber forest, and then a turn will be performed immediately. As shown in Figure 3, the green circles represent trees; black points stand for the ideal stopping spots; and the red dotted line is regarded as the robot’s ideal navigation path.

### 2.3. Navigation Phases

According to the data applied to the robot’s navigation and the corresponding movements, the navigation is divided into three phases: “straight phase”, “turning phase”, and “row-changing phase”.

As shown in Figure 3, at the A-B stage of the straight phase, the sparse point cloud data of tree trunks are extracted based on data offered by LiDAR and the gyroscope. Then, the point cloud is fitted though the Gauss–Newton method to obtain the coordinates of each tree’s center. The straight line connecting tree centers is fitted by the Least Squares method and then regarded as the robot’s navigation path. According to the navigation path, the robot would walk along one row with a fixed lateral distance and stop at the designated spot in front of each tree.

At the B-C stage of the turning phase, after finishing the rubber tapping at point B, the robot will walk straight to point C (the distance between B and C is controlled by setting the straight-walking time). Then, the data of the gyroscope’s Z-axis and differential driving are employed to make a 90-degree turn.

At the C-D and E-F stages of the row-changing phase, the navigation of C-D is similar to that of the “straight phase”. Their difference lies in that C-D has no fixed-point stopping, and it will use the gyroscope to turn right again when it goes through a complete row space. The E-F stage is similar to the B-C stage: when finishing the tapping, the robot will go straight toward point E, make a 90-degree left turn with the help of the gyroscope, walk straight to point F (the distance of E-F is controlled by setting the straight-walking time), make another 90-degree left turn using the gyroscope, and enter the next cycle.

The above three phases constitute the whole cycle of the robot’s navigation. When the robot encounters situations beyond the above three phrases, it will stay put. On one hand, this can ensure the safety of the robot. On the other hand, after finishing all of the operations in the rubber forest, it can stop automatically.

### 2.4. Calibration of the Installed Location of LiDAR

**Figure 4:**
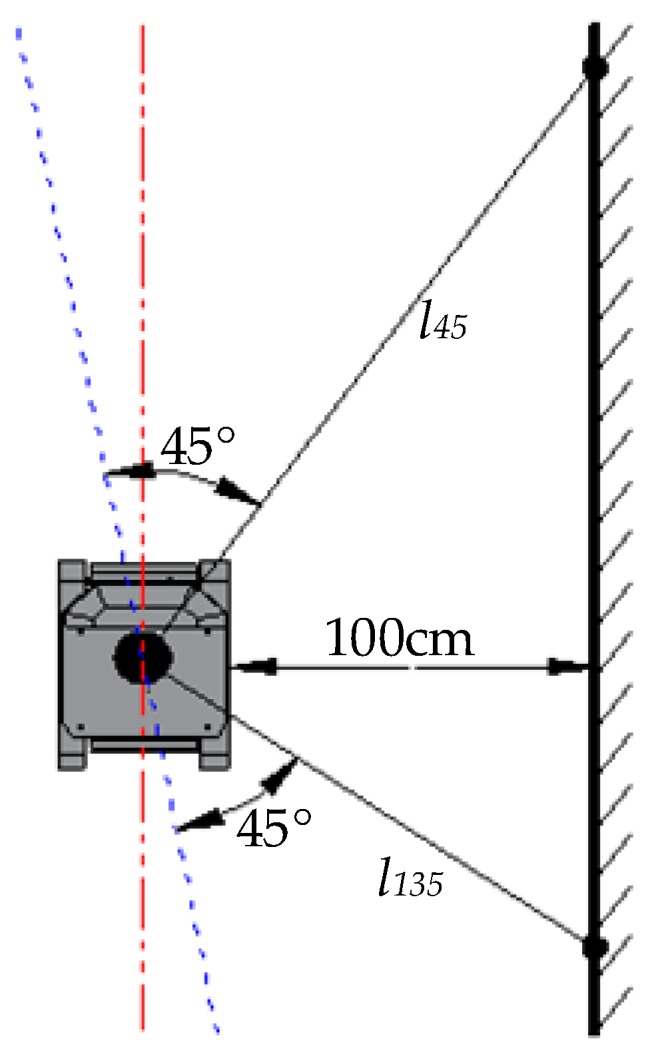
Calibration of the LiDAR’s installed location.

It is important to calibrate the installed location of the vehicular LiDAR [27]. Whether the axis of the LiDAR, i.e., the 0–180° axis, is parallel to the center line of the robot platform or not directly influences the navigating performance of the robot and the accuracy of information collection. When installing, the vertical wall is utilized to calibrate the center line of the LiDAR, so as to make the center line of the LiDAR parallel to the center line of the robot. As shown in Figure 4, the robot is put at the place 100 cm away from the wall and the center line of the robot is parallel to the wall. Then, on the wall, two points that can form the 45° and 135° angles are extracted from the LiDAR’s scanned data, and the distance between these two points and the Lidar’s central point are set as *l_45_* and *l_135_*, respectively. Finally, the LiDAR’s installation position should be calibrated until *l_45_ = l_135_*.

### 2.5. Navigation Path Generation

Firstly, all scanned data are sorted according to angles, and then data within 0~180°, i.e., the robot’s right-side data are extracted. In order to make full use of the tree data closest to the robot and to fit the tree row better, the LiDAR scanning distance is set to be twice the maximum plant spacing. All scanned points on the tree are extracted using the clustering method [31,33]. If two points meet the following requirements, they fall into the same category:

(1) The distance difference between two points and the LiDAR is less than the threshold *δ_1_* (*δ_1_* is determined by the diameter), i.e.,
  $$\left|l_{1}-l_{2}\right|<\delta_{1};$$
(2) The angle difference between two points and the LiDAR is less than the threshold *δ_2_* (*δ_2_* is determined by the diameter and the distance between the robot and the tree row), i.e.,
  $$\left|\alpha_{1}-\alpha_{2}\right|<\delta_{2};$$
(3) The distance difference between two points and the center line of the robot is less than the threshold *δ_3_* (*δ_3_* is determined by the row spacing), i.e.,
  $$\left|l_{1}sin\alpha_{1}-l_{2}sin\alpha_{2}\right|<\delta_{3}.$$

After clustering, if the number of elements in a cluster is larger than the given threshold *N* *(N* is determined by the number of points scanned on the tree with the smallest diameter), it can represent a tree:

*Num(class_i_) > N* ➞ *class_i_ = TreeTrunk*.

In the same way, other tree trunk information of the nearest tree row from the robot can be obtained. If there are small trees or stumps that do not need to be operated on, the robot can automatically ignore them by setting the threshold *N*. All data used in the navigation come from the tree row closest to the robot, so different row spacings and plant spacings in the forest are permitted.

Due to the uneven terrain in the forest, slight tilt may occur. This will lead to an increase in the distance error. Therefore, the gyroscope should acquire attitude information of the robot in real time to correct the LiDAR data based on the roll and pitch angles.

**Figure 5:**
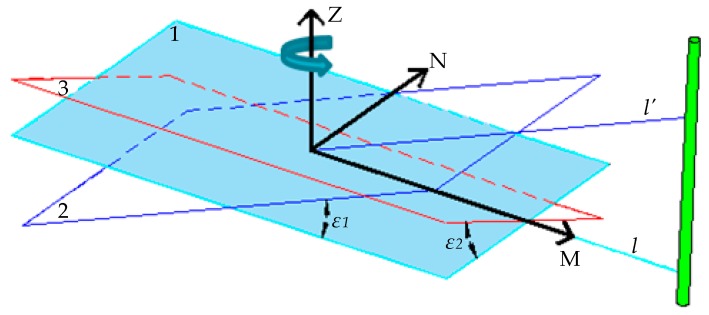
Attitude compensation of the robot.

As shown in Figure 5, plane 1 is horizontal, and the attitude information of the robot is acquired in real time by the gyroscope, including the left and right tilt angle *ε_1_*, the front and rear tilt angle *ε_2_* to plane 1, and the rotation angle around the Z axis. The tilt angle is utilized to compensate for the scanning distance error of the LiDAR when the robot walks on uneven ground. When the robot tilts to the left or right, *l* and *l’* represent the scanning distance of the LiDAR on plane 1 and plane 2, respectively, while the cylinder represents the tree. The actual distance between the robot and the tree is:

$$l=l^{\prime }cos\varepsilon_{1}$$

Similarly, when the robot tilts to the front or rear, the actual distance between the robot and the tree is:

$$l=l^{\prime }cos\varepsilon_{2}$$

When the robot has both left–right tilt and front–rear tilt, the total tilt angle of the robot should be the combination of these two parts:

$$l=l^{\prime }cos\varepsilon_{1}cos\varepsilon_{2}$$

**Figure 6:**
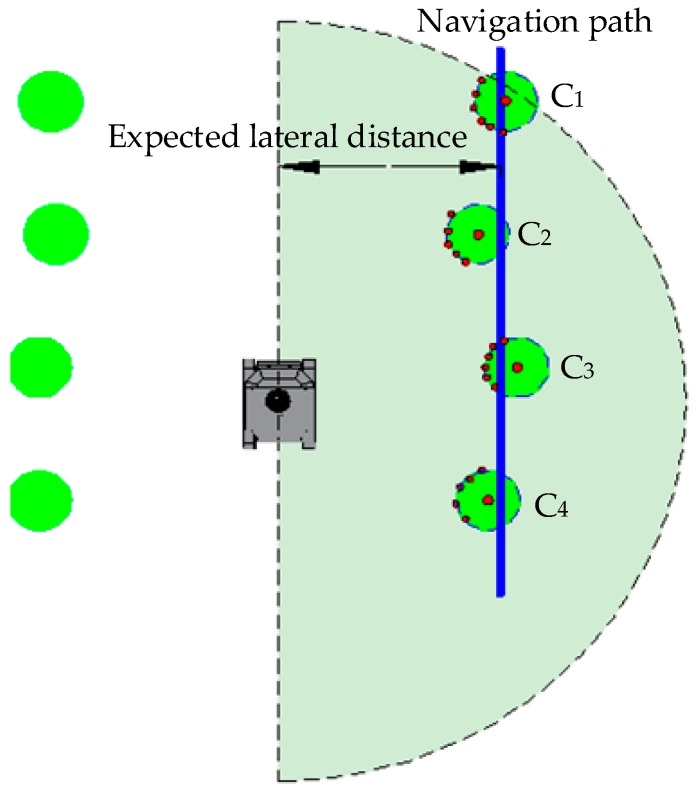
Fitting the navigation path.

After the clustering is completed, all point cloud data on the trunk for navigation can be extracted then round-fitted through the "Gauss–Newton method" to obtain the center coordinates of C_1_, C_2_, C_3_, and C_4_, as shown in Figure 6. Then, the Least Squares method is adopted to fit the straight line of the center, which shall be the navigation path. 

### 2.6. Design of the Fuzzy Controller

The advantage of Fuzzy Control [34] is that it does not need to establish an accurate control model. We only need to define effective input and output control variables and appropriate Fuzzy Control rules [35]. In this paper, the lateral error E and the heading error θ are used as the input of the Fuzzy Controller. The forward direction of the robot is taken as the reference. If E is positive, it means that the robot’s position deviates from the left side of the ideal navigation path. If E is negative, it means that the robot position deviates from the right side of the ideal navigation path. At the same time, the positive heading angle error indicates the counterclockwise deflection, and the negative angle indicates the clockwise deflection.

**Figure 7:**
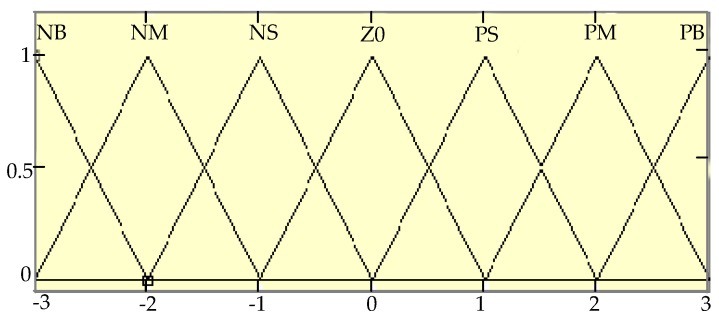
The distribution of the membership function of θ, E, and U.

The PWM (Pulse-Width Modulation) control difference, U, of the DC motors on both the left and the right sides is selected as the output variable, so as to control the differential steering of the robot. The heading angle error θ, lateral error E, and output quantity U are divided into seven levels: negative big (NB), negative medium (NM), negative small (NS), zero (Z0), positive small (PS), positive medium (PM), and positive big (PB). The basic fields of θ, E, and U are respectively [−6°, 6°], [−250 cm, 250 cm], [−120, 120]. Their corresponding fuzzy fields are all {−3, −2, −1, 0, 1, 2, 3}. The membership function adopts a triangle. Then, the distribution of this fuzzy variable membership function can be seen in the following Figure 7.

**Figure 8:**
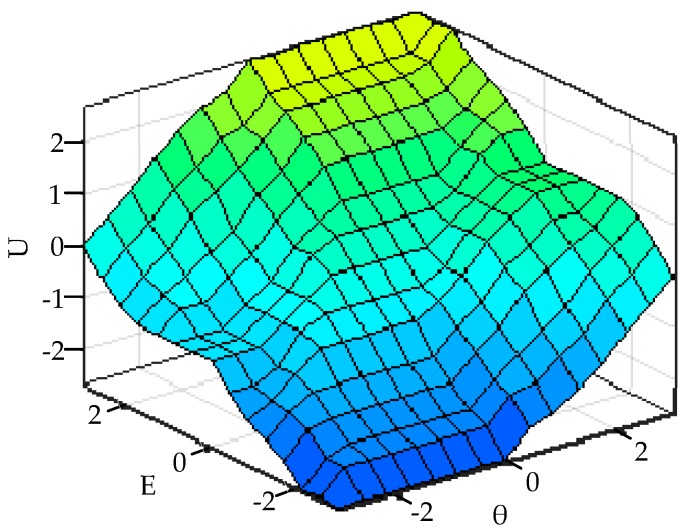
The surface schematic diagram of the fuzzy control rules.

The MIN-MAX-gravity method is selected for defuzzification. According to experience and the test conditions, the table of fuzzy control rules is shown in Table 1. Figure 8 shows the three-dimensional surface diagram of the fuzzy control rules.

### 2.7. Location of the Robot

The feature-based Extended Kalman Filter (EKF) [36] provides an effective method for mobile robot pose estimation, so it was adopted in this study for robot localization in the forest. It is stipulated that, when the robot stops in front of each tree, the exact spot is regarded as the initial position of the robot, *(x’_0_, y’_0_)*. The pose of the robot in the forest can be expressed by its coordinates *(x’, y’)* and the attitude angle φ relative to the trunk. Assuming that the robot is moving at a constant speed between the two trees, the pose of the robot should be:

{x′(k+1)=x′(k)+ux′(k)Ty′(k+1)=y′(k)+vy′T+12uy′(k)T2vy′(k+1)=vy′(k)+uy′(k)Tφ(k+1)=φ(k)+uφ(k)T

In the above formulas, *T* is the sampling time; *u(k)* the random perturbation in motion; and *v_y’_* the speed of the car in the *y’*-axis direction. 

For sake of convenience, the equation of the state of the robot system should be:

(1) $${\hat{x}}_{k}^{-}=f(x_{k-1}^{\prime },y_{k-1}^{\prime },\overset{•}{y_{k-1}^{\prime }},\varphi_{k-1})$$

Then, the error covariance matrix should be:

(2) $$P_{k}^{-}=A_{k}P_{k-1}A_{k}^{T}+W_{k}Q_{k}W_{k}^{T}$$

In the above formula, $A_{k}=\frac{\partial f}{\partial X}$ stands for the Jacobian matrix of the system state, $W_{k}=\frac{\partial f}{\partial U}$ the Jacobian matrix of process noise, and *Q_k_* the covariance matrix of process noise.

The Kalman Gain can be calculated as:

(3) $$K_{k}=P_{k}^{-}H_{k}^{T}{\left(H_{k}P_{k}^{-}H_{k}^{T}+V_{k}R_{k}V_{k}^{T}\right)}^{-1}$$

In the above formula, $H_{k}=\frac{\partial h}{\partial X}$ is the Jacobian matrix for measuring the state of the model, $V_{k}=\frac{\partial h}{\partial U}$ the Jacobian matrix for measuring model noise, and *R_k_* the covariance matrix of process measurement noise.

Since the robot’s initial position *(x‘_0_, y’_0_)* is already known, the position at time k should be *(x‘(k), y’(k))*. Distances between the tree and the LiDAR can be measured by the LiDAR, while the heading angle of the robot can be measured by the gyroscope. Therefore, the measurement model should be:

$$h(x^{\prime }{}_{k},y^{\prime }{}_{k},\varphi_{k})=\left[\begin{array}{c} r_{k}\\ \phi_{k} \end{array}\right]=\left[\begin{array}{c} \sqrt{{\left(x^{\prime }(k)-x^{\prime }{}_{0}\right)}^{2}+{\left(y^{\prime }(k)-y^{\prime }{}_{0}\right)}^{2}}\\ \arctan(\frac{y^{\prime }(k)-y^{\prime }{}_{0}}{x^{\prime }(k)-x^{\prime }{}_{0}})+\varphi_{k} \end{array}\right]$$

Using actually measured data to correct the robot’s attitude estimation:

(4) $${\hat{x}}_{k}={\hat{x}}_{k}^{-}+K_{k}(Z_{k}-h({\hat{x}}_{k}^{-}))$$

In the above formula, *Z_k_* stands for the actually measured data.

Finally, the error covariance matrix is updated as follows:

(5) $$P_{k}=(I-K_{k}H_{k})P_{k}^{-}$$

**Figure 9:**
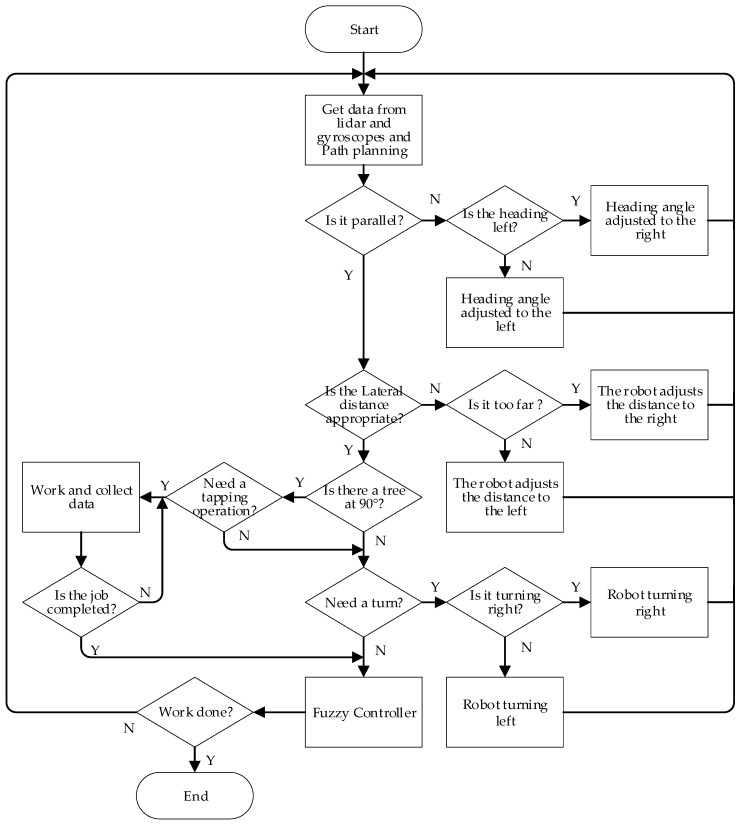
The flow chart of navigation control.

Extended Kalman Filter (EKF) is a recursive estimation process, and the updated pose and error covariance matrix are used to predict the new estimates in the next time step. Figure 9 is the flow chart of the system algorithm.

### 2.8. Information Collection

The information collected on the forest includes tree diameters, plant spacing, row spacing, and position information, among which tree position derives from plant spacing and row spacing recursively. The specific methods can be seen as follows.

#### 2.8.1. Calculation of Tree Position

**Figure 10:**
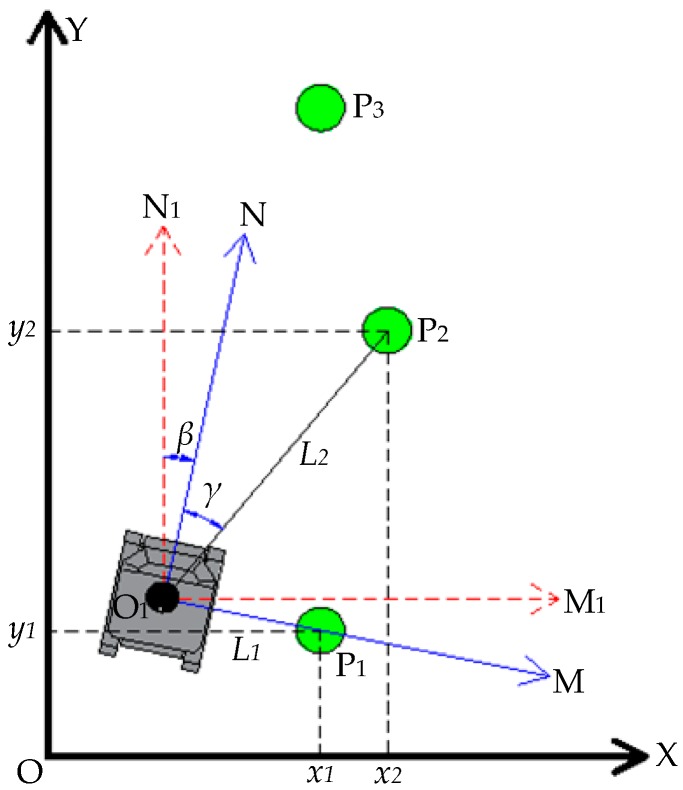
The transformation of a tree’s coordinates.

As shown in Figure 10, the absolute coordinate system XOY is established according to the position of tree P_1_ and the trend of the entire forest. The absolute coordinate of tree P_1_ is *(x_1_, y_1_)*. When the robot stops in front of tree P_1_ to carry out the operation, the center of its LiDAR is taken as the origin point O_1_. Then, the dead-ahead direction of the robot is taken as the N-axis, and the line connecting O_1_ and the tree is taken as the M-axis, so as to establish the robot’s coordinate system MO_1_N. The distance between P_1_ and O_1_ is *L_1_*; the distance between P_2_ and O_1_ is *L_2_*; and the angle between P_2_ and the N-axis is *γ*. The coordinate system MO_1_N is rotated by *β* degrees (counterclockwise is positive; the *β* angle is measured by the gyroscope), so that the newly obtained coordinate system M_1_O_1_N_1_ is parallel with the absolute coordinate system XOY. Thus, the coordinate of tree P_2_ in the M_1_O_1_N_1_ coordinate system should be:

$$\left(L_{2}sin(\gamma +\beta ),L_{2}cos(\gamma +\beta )\right)$$

The coordinate of P_2_ in the absolute coordinate system XOY should be:

$$\left[\begin{array}{c} x_{2}\\ y_{2} \end{array}\right]=\left[\begin{array}{c} x_{1}+L_{2}sin(\gamma +\beta )-L_{1}cos\beta \\ y_{1}+L_{2}cos(\gamma +\beta )+L_{1}sin\beta \end{array}\right]$$

Therefore, the absolute coordinate of tree P_3_ can be deduced from the absolute coordinates of tree P_2_. In the same way, the coordinate of all trees in the same row in the absolute coordinate system can be calculated, and coordinate calculation formulas for different rows can be inferred. Tree rows in the actual experiment are so straight that the X-coordinate of each row is regarded as the same. The coordinate formula of tree position in the forest is obtained as follows:

The coordinate formula of odd tree rows should be:

$$\left[\begin{array}{c} x_{1n}\\ y_{1n} \end{array}\right]=\left[\begin{array}{c} x_{11}\\ y_{11}+\sum_{i=1}^{n-1}\left(L_{i+1}cos(\gamma_{i}+\beta_{i})+L_{i}sin\beta_{i}\right) \end{array}\right].$$

The coordinate formula when the robot turns:

$$\left[\begin{array}{c} x_{21}\\ y_{21} \end{array}\right]=\left[\begin{array}{c} x_{1n}+L_{2}cos(\gamma_{2}+\beta )-L_{1}cos(\gamma_{1}+\beta )\\ y_{1n}+L_{2}sin(\gamma_{2}+\beta )-L_{1}sin(\gamma_{1}+\beta ) \end{array}\right].$$

The coordinate formula of even tree rows should be:

$$\left[\begin{array}{c} x_{2n}\\ y_{2n} \end{array}\right]=\left[\begin{array}{c} x_{21}\\ y_{21}-\sum_{i=1}^{n-1}\left(L_{i+1}cos(\gamma_{i}+\beta_{i})-L_{i}sin\beta_{i}\right) \end{array}\right].$$

#### 2.8.2. Calculation of Tree Radius

In order to improve the accuracy of the measurement of a tree’s diameter as much as possible, when the robot stops in front of the tree for operation, the LiDAR’s position is closest to the tree and the robot pauses. At this time, the scanned point set of this tree and the coordinate of the next tree’s relative position are collected and circularly fitted using the Gauss–Newton method [37]. Thus, the diameter of the obtained circle is regarded as the diameter of the tree.

The curvilinear equation of the circular is supposed to be:

$$x^{2}+y^{2}+ax+by+c=0.$$

*C, D, E, G*, and *H* are assumed as follows:

$$\{\begin{array}{l} \begin{array}{l} C=N\sum x_{i}^{2}-{\left(\sum x_{i}\right)}^{2}\\ D=N\sum (x_{i}y_{i})-\sum x_{i}\sum y_{i} \end{array}\\ E=N\sum x_{i}^{3}+N\sum (x_{i}y_{i}^{2})-\sum (x_{i}^{2}+y_{i}^{2})\sum x_{i}\\ G=N\sum y_{i}^{2}-{\left(\sum y_{i}\right)}^{2}\\ H=N\sum y_{i}^{3}+N\sum (x_{i}^{2}y_{i})-\sum (x_{i}^{2}+y_{i}^{2})\sum y_{i} \end{array}$$

Then *a, b,* and *c* should be:

$$\{\begin{array}{l} a=\left(HD-EG\right)/\left(CG-D^{2}\right)\\ b=\left(HC-ED\right)/\left(D^{2}-GC\right)\\ c=-(\sum (x_{i}^{2}+y_{i}^{2})+a\sum x_{i}+b\sum y_{i})/N \end{array}$$

Thus, the tree’s radius *R* should be:

$$R=1/2\sqrt{a^{2}+b^{2}-4c}.$$

After obtaining each tree’s position and diameter according to the above method, in order to help users have a better understanding of the collected information, the position of the tree is embodied by the position of the circle’s center and the diameter of the tree by the diameter of the circle. The geometric feature map of the forest is thus graphed.

## 3. Results

### 3.1. Navigation Results Analysis

**Figure 11:**
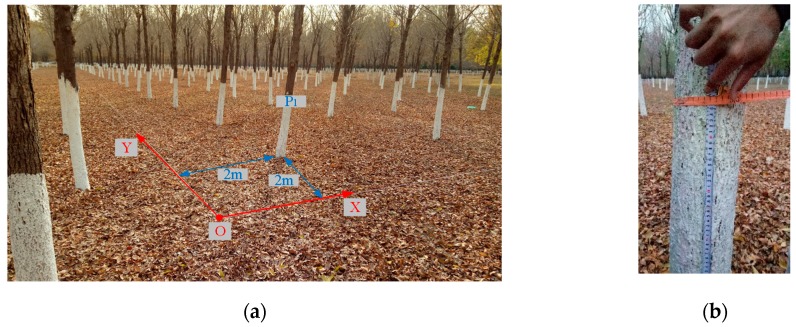
Actual parameter measurement in the test forest. (**a**) Position measurement. (**b**) Circumference measurement.

In order to test the navigation performance and the accuracy of information collection of the LiDAR–gyroscope-based navigation system, a forest about 800 m^2^ in area is selected as the test area south of the Agricultural Science and Technology Park in Tongzhou District, Beijing (116° 49’ 13’’ E, 39° 51’ 29’’ N). This test area is composed of three rows of trees, with 15 trees in each row, so there are 45 trees altogether. As shown in Figure 11a, the absolute coordinate system XOY is established by taking tree P_1_ as the reference, with moving backward and left 2 m as the origin O. In the absolute coordinate system, the actual position of the tree is measured by hand with a tape measure. Then, we can obtain the plant spacing and row spacing. In Figure 11b, the circumference of the trunk at the same height as the LiDAR (95 cm from the ground) was measured with a tape measure in the test forest, and data on the diameter of the tree were obtained. The specific parameters are shown in Table 2. Because the distance between the test forest and adjacent forests is 1000 cm, to guarantee the integrity of this test and make the robot turn at the boundaries, the scanning radius of the LiDAR was set to be 900 cm, and the initial speed 0.3 m/s.

In this test, white wheat flour is placed in the tracing device, so, when the robot walks, the white line left on the ground should be the actual walking path of the robot. The heading direction of the robot is regarded as “front”, and the difference between the actual stopping site and the ideal site is regarded as the “front and back error”, just as what is shown by the yellow dot in Figure 3. If the robot stops in front of the ideal stopping site, the error is positive. If the robot stops at the back of the ideal stopping site, the error is negative.

**Figure 12:**
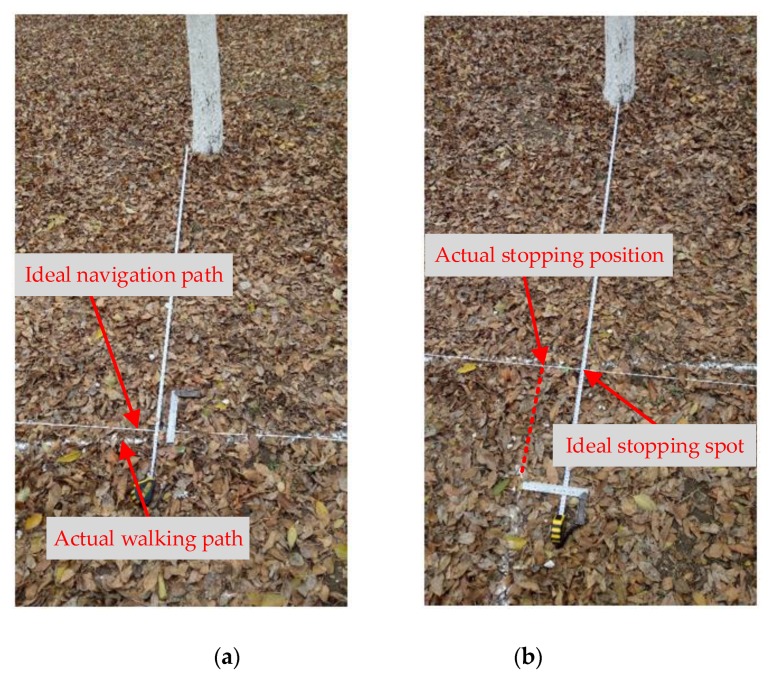
Error measurement. (**a**) Lateral error measurement; (**b**) Front and back error measurement.

**Figure 13:**
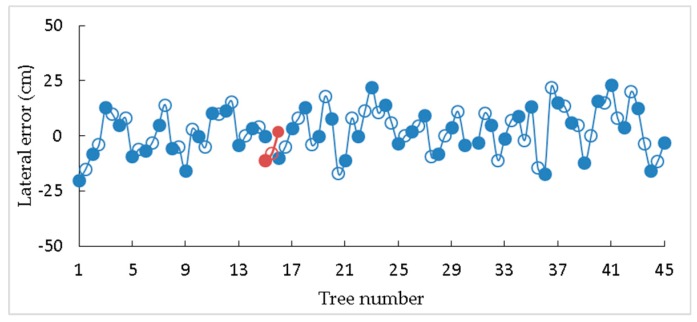
The change in lateral error in the second test.

Three repeated tests have been conducted in this forest. When measuring lateral errors, the points at 125 cm in front of each tree are connected in turn as the ideal navigation path for the robot. In the test, white wheat flour is placed in the tracing device, so, when the robot walks, the white line left on the ground should be the actual walking path of the robot. As shown in Figure 12a, the lateral error is measured between the ideal navigation path and the actual walking path of the robot. When the robot stops in front of the tree (the robot stops for 3 seconds), the position of the LiDAR relative to the tree is marked with white wheat flour. As shown in Figure 12b, the distance between the actual and ideal stopping spots is measured as the front and back errors. After each experiment, the lateral error data were sampled in front of each tree (data are represented by solid circles in Figure 13) and in front of the midpoint of the connection between two trees (data are represented by hollow circles in Figure 13). The front and back error data were sampled in front of each tree. 

Table 3 shows the result of lateral errors when the robot walked along one row and turned at the end of the row, while Table 4 shows the result of the front and back errors. Figure 13 suggests the lateral error of the robot at each separate measurement point in the second test. The solid blue line represents the lateral error when the robot walked along one row, and the red dotted line represents the lateral error when turning. The root mean square (RMS) lateral errors with the three repeated tests are 10.31 cm, 10.32 cm, and 9.37 cm, respectively, which shows that the method has great path tracking performance. In the three repeated tests, the average values of the front and back errors are all positive, mainly because the inertia makes the robot move forward for a distance after receiving the stop command.

In order to describe the robot’s stopping range around each tree, the stopping error is prescribed as:

$$StoppingError=\sqrt{{\left(LateralError\right)}^{2}+{\left(FrontAndBackError\right)}^{2}}.$$

**Figure 14:**
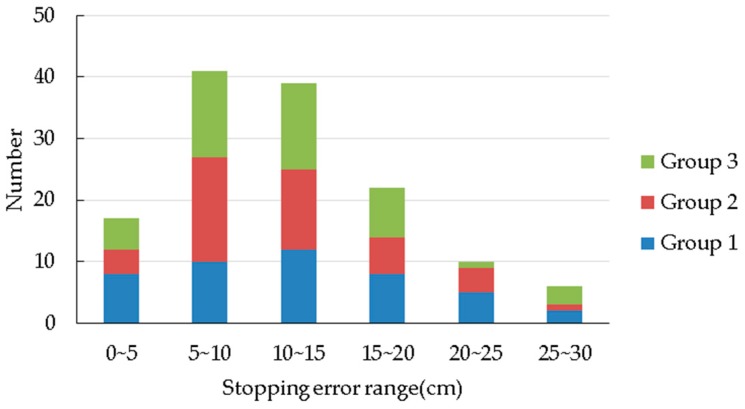
Stopping error distribution.

Table 5 shows the result of the stopping errors. In Figure 14, the abscissa represents the range of stopping errors, and the ordinate represents the number of data elements within the range of errors. Figure 14 suggests that the stopping error follows a normal distribution. Most of the errors are within the range of 5~15 cm, and the average stopping error is 12.08 cm, which meets the requirements of forest navigation for a rubber-tapping robot.

### 3.2. Mapping Results Analysis

**Figure 15:**
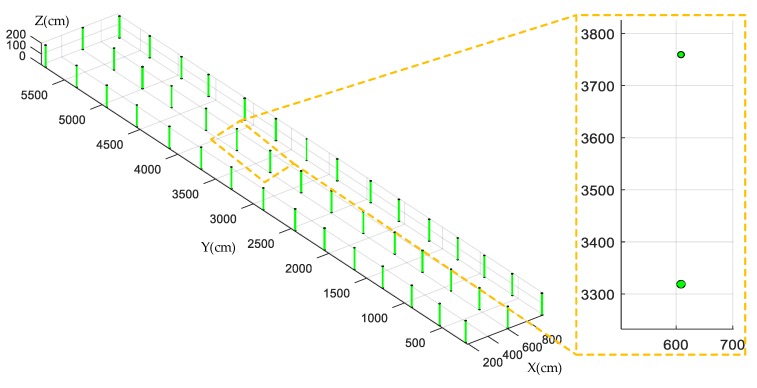
The geometric feature map of the forest.

In order to help users have a better understanding of the collected information, a geometric feature map [38] of the test forest is constructed to show the plant spacing, row spacing, diameter, and position of trees. On the basis of the above results, the point set of each tree is fitted into a circle by the "Gauss–Newton method". According to the above method of measuring plant spacing, row spacing, tree position, and tree diameter as well as the method of graphing a geometric feature map, the position of a tree is represented by the center of the circle, and the diameter of the tree by the diameter of the circle. The map of the forest and an enlarged partial detail can be seen in Figure 15.

To evaluate the accuracy of the constructed geometric feature map, the tree’s position error *P_e_* is defined as follows:

$$P_{e}=\sqrt{{\left(x_{t}-x_{i}\right)}^{2}+{\left(y_{t}-y_{i}\right)}^{2}}.$$

In the above formula, (x_t_, y_t_) stands for the actual position of the tree, and (x_i_, y_i_) the calculated position of the tree according to collected data. The tree’s radius error *r_e_* is defined as follows:

$$r_{e}=r_{i}-r_{t}.$$

In the above formula, *r_t_* represents the actual radius of the tree while *r_i_* represents the calculated radius of the tree according to collected data. Similarly, the plant spacing error *h_e_* is defined as follows:

$$h_{e}=h_{i}-h_{t}.$$

Table 6 shows the position error, radius error, and plant spacing error of trees.

**Figure 16:**
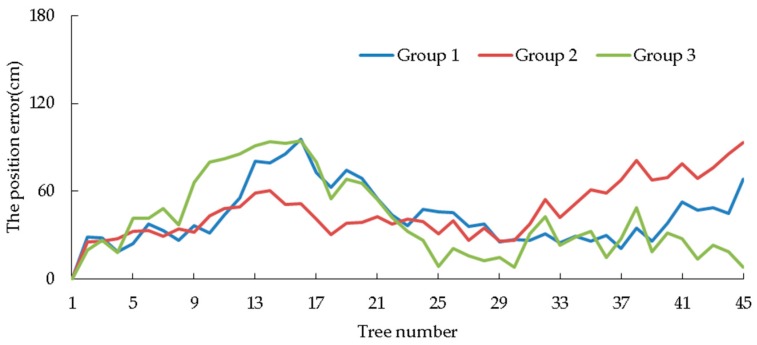
The position error of the tree.

**Figure 17:**
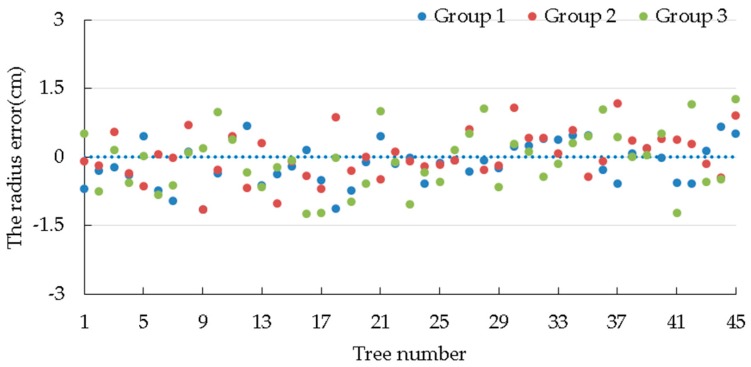
The radius error of the tree.

Figure 16 and Figure 17 show the position error and radius error of trees, respectively. After analyzing Table 6, it can be seen that all average values of plant spacing errors in these three tests are negative, which indicates that the tree distance measured by the robot is shorter than the actual condition on the whole. This is mainly because the robot is regarded as stopping at the ideal position by the default setting when measuring tree spaces, just as what the black dot shows in Figure 3. Table 4 shows that the average front and back error in these three tests is positive and similar to the average value of the plant spacing error. It shows that, due to the inertia of the robot, where the robot stops is generally in front of the ideal spot, just as what is shown by the yellow dot in Figure 3. Therefore, the measured plant spacing is shorter than the actual condition.

**Figure 18:**
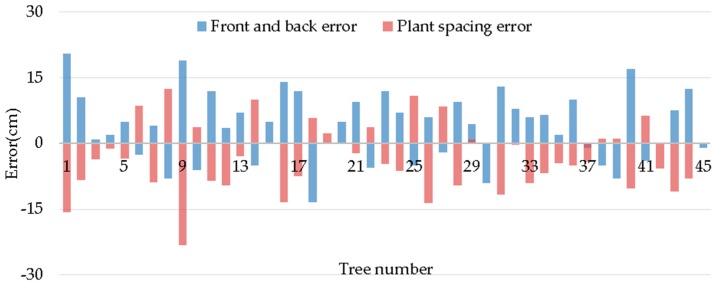
Comparisons of the front and back error and the plant spacing error.

Figure 18 shows a comparison between the front and back errors and the plant spacing errors in the second test. It can be seen that the front and back error and the plant spacing error are generally distributed symmetrically, which proves the previous inference to be right. This indicates that the plant spacing error mainly derives from the front and back errors.

As shown in Figure 16, from the first tree to the fifteenth tree, i.e., in the first tree row, the tree position error increases gradually. This is mainly because the tree position is the recursion of plant spacing; a smaller plant spacing leads to a plant spacing error, the accumulation of which leads to the growth of the tree position error. From the 16th tree to the 30th tree, i.e., in the second tree row, when the robot returns, a part of the error will be compensated for, so the tree position error decreases gradually. However, in the third tree row, the tree position error gradually increases again.

Figure 17 shows that the radius error of the tree fluctuates within a small range around 0, and the RMS radius errors are 0.49 cm, 0.51 cm, and 0.66 cm, respectively, which shows that the measured radius reflects the true radius of the tree very well. The tree radius error is mainly caused by longitudinal cracks in trees and the unevenness of the ground. Among the information collected on the forest during the tests, the maximum radius error is 1.26 cm, while the maximum plant spacing error is 26.19 cm. The results show that the collected data could reflect the true parameters of the forest well.

**Figure 19:**
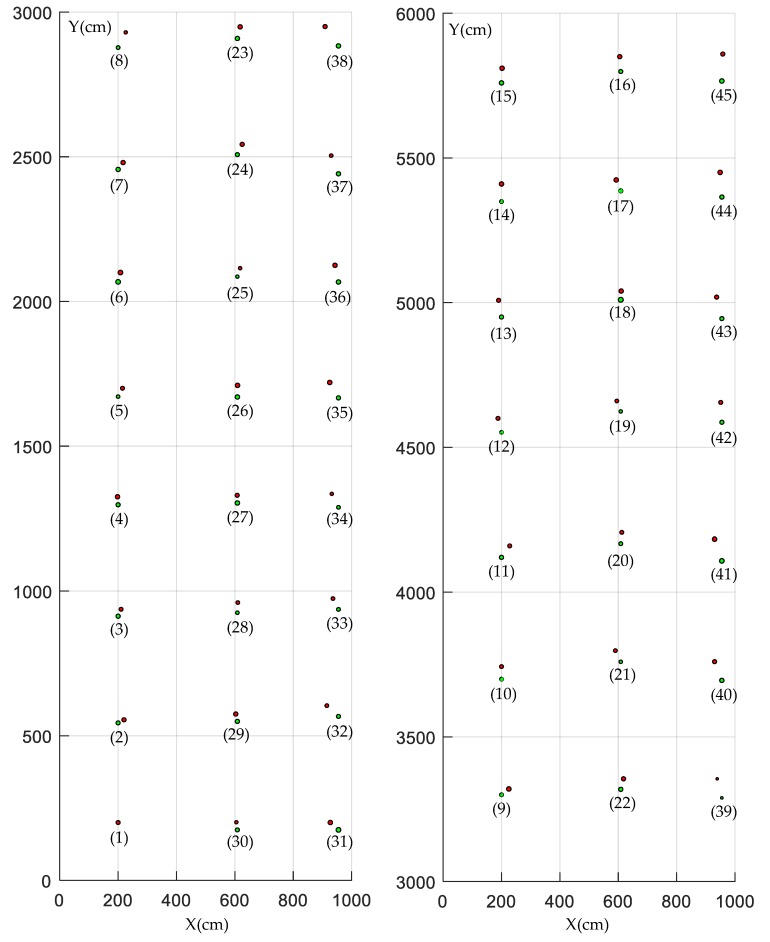
Comparison of forest maps.

Figure 19 shows a comparison between the actual map of the forest and the graphed map. In this figure, the red stands for the actual geometric feature map, the green stands for the constructed geometric feature map, and the number in parentheses is the tree number. In the constructed geometric feature map, the average position errors are 43.00 cm, 46.48 cm, and 40.40 cm, respectively, indicating that the map containing information on the forest not only benefits the informatization and precision management of trees, but also provides references for navigating a robot based on prior maps.

## 4. Conclusions

1. A method for forest navigation for an automatic rubber tapping platform was proposed in this paper. Instead of relying on GPS and prior maps, the method makes the robot walk along one row at a fixed lateral distance, stop at a fixed point, and turn from one row into another, only using low-cost two-dimensional (2D) LiDAR and a gyroscope. The root mean square (RMS) lateral errors with three repeated tests are 10.31 cm, 10.32 cm, and 9.37 cm, respectively, which shows that the method has great path tracking performance. The method provides better fixed-point stopping with average stopping errors of 12.62 cm, 11.98 cm, and 11.64 cm, respectively, which meets the requirements of forest navigation for a rubber-tapping robot.
2. A method of collecting forest information in real time during navigation was developed. Among the information collected on the forest during the tests, the RMS radius errors are less than 0.66 cm, and the RMS plant spacing errors less than 11.31 cm. The results show that the collected data could reflect the true parameters of the forest well.
3. A method for constructing a geometric feature map based on the collected information was introduced. In the constructed geometric feature map, the average position errors are 43.00 cm, 46.48 cm, and 40.40 cm respectively, indicating that the map containing information on the forest not only benefits the informatization and precision management of trees, but also provides references for navigating a robot based on prior maps.

## Figures and Tables

**Table 1:** The fuzzy control rules.

U		E						
		NB	NM	NS	Z0	PS	PM	PB
θ	NB	NB	NB	NM	NS	NS	NS	Z0
	NM	NB	NM	NS	NS	NS	Z0	PS
	NS	NB	NM	NS	Z0	Z0	PS	PM
	Z0	NB	NM	NS	Z0	PS	PM	PB
	PS	NM	NS	Z0	Z0	PS	PM	PB
	PM	NS	Z0	PS	PS	PS	PM	PB
	PB	Z0	PS	PS	PS	PM	PB	PB

**Table 2:** The parameters of the tested forest.

Category	Parameter	Average	Standard Deviation
Plant Spacing Range /cm	348.5~490.7	402.8	26.6
Diameter Range /cm	8.4~15.9	13.8	1.4
1, 2 row spacing /cm	405.0	/	/
2, 3 row spacing /cm	322.0	/	/
1st row length /cm	5810	/	/
2nd row length /cm	5850	/	/
3rd row length /cm	5859	/	/

**Table 3:** Lateral error.

Test Number		1	2	3
Walking along one row (cm)	Maximum	29.0	23.0	27.0
	Average	2.25	2.29	−0.78
	Standard Deviation	10.07	10.06	9.34
	RMS	10.31	10.32	9.37
Turning (cm)	Maximum	14.0	−11.0	9.0
	Average	4.33	−5.67	2.67
	Standard Deviation	10.34	5.56	8.26
	RMS	11.21	7.94	8.68

**Table 4:** Front and back error.

Test Number	1	2	3
Maximum /cm	25.0	20.5	27.0
Average /cm	4.10	3.91	3.74
Standard Deviation /cm	9.53	7.82	9.13
RMS/cm	10.37	8.74	9.87

**Table 5:** Stopping error.

Test Number	1	2	3
Maximum /cm	27.73	28.64	28.79
Average /cm	12.62	11.98	11.64
Standard Deviation /cm	6.51	6.45	6.30

**Table 6:** Map errors.

Test Number		1	2	3
Position error (cm)	Maximum	95.61	93.19	94.64
	Average	43.00	46.48	40.40
	Standard Deviation	20.02	19.06	26.96
Radius error (cm)	Maximum	−1.15	1.16	1.26
	Average	−0.13	0.03	−0.06
	Standard Deviation	0.47	0.51	0.66
RMS		0.49	0.51	0.66
Plant spacing error (cm)	Maximum	−26.19	−23.16	−25.89
	Average	−4.47	−3.41	−4.23
	Standard Deviation	10.51	7.80	10.28
RMS		11.31	8.43	11.00
